# Application of an Antibacterial Coating Layer *via* Amine-Terminated Hyperbranched Zirconium–Polysiloxane for Stainless Steel Orthodontic Brackets

**DOI:** 10.1049/2024/4391833

**Published:** 2024-02-12

**Authors:** Yaxin Qu, Xinwei Lu, Tingting Zhu, Jie Yu, Zhe Zhang, Yu Sun, Yuanping Hao, Yuanfei Wang, Yanling Yu

**Affiliations:** ^1^Department of Stomatology, School of Stomatology of Weifang Medical University, Weifang 261053, China; ^2^School of Stomatology of Qingdao University, Qingdao 266003, China; ^3^Qingdao Stomatological Hospital Affiliated to Qingdao University, Qingdao 266001, China

## Abstract

The massive growth of various microorganisms on the orthodontic bracket can form plaques and cause diseases. A novel amine-terminated hyperbranched zirconium–polysiloxane (HPZP) antimicrobial coating was developed for an orthodontic stainless steel tank (SST). After synthesizing HPZP and HPZP-Ag coatings, their structures were characterized by nuclear magnetic resonance spectroscopy, scanning electron microscopy, thickness measurement, contact angle detection, mechanical stability testing, and corrosion testing. The cell toxicity of the two coatings to human gingival fibroblasts (hGFs) and human oral keratinocytes (hOKs) was detected by cell counting kit eight assays, and SST, HPZP@SST, and HPZP-Ag@SST were cocultured with *Staphylococcus aureus*, *Escherichia coli*, and *Streptococcus mutans* for 24 hr to detect the antibacterial properties of the coatings, respectively. The results show that the coatings are about 10 *μ*m, and the water contact angle of HPZP coating is significantly higher than that of HPZP-Ag coating (*P* < 0.01). Both coatings can be uniformly and densely distributed on SST and have good mechanical stability and corrosion resistance. The cell counting test showed that HPZP coating and HPZP-Ag coating were less toxic to cells compared with SST, and the toxicity of HPZP-Ag coating was greater than that of HPZP coating, with the cell survival rate greater than 80% after 72 hr cocultured with hGFs and hOKs. The antibacterial test showed that the number of bacteria on the surface of different materials was ranked from small to large: HPZP@SST < HPZP-Ag@SST < SST and 800 *μ*g/mL HPZP@SST showed a better bactericidal ability than 400 *μ*g/mL after cocultured *with S. aureus*, *E. coli*, and *S. mutans*, respectively (all *P* < 0.05). The results showed that HPZP coating had a better effect than HPZP-Ag coating, with effective antibacterial and biocompatible properties, which had the potential to be applied in orthodontic process management.

## 1. Introduction

Stainless steel orthodontic brackets are one of the most widely used aligners for orthodontic treatment [1, 2], and their complex, uneven structure and prolonged intraoral bonding provide conditions for plaque retention, which affects the maintenance of oral hygiene [3–5]. Studies have shown that the number of *Streptococcus pyogenes* and *Lactobacillus* spp. increases significantly during orthodontic treatment, and white spot lesions on the tooth surface can occur in 2%–96% of patients [6, 7], progressing to dental caries, gingivitis [8], and periodontitis [9, 10]. Developing an antibacterial and biocompatible coating material has crucial therapeutic implications for avoiding problems during orthodontic procedures [11–13].

To date, a combination of different types of antibacterial coating materials and scaffolds has been developed, such as organic antibacterial materials, quaternary amine salts [14–18], inorganic antibacterial materials (metals, metal oxides, etc.) [19–21], natural antimicrobial materials, and composite antimicrobial materials [22]. Łyczek et al. [23] embedded gold nanoparticles (AuNPs) in a polyoxoborate matrix to form a new inorganic nanocomposite, BOA (BOA: B-boron, O-oxygen, A-gold, Latin aurum), by immersing the brackets in a colloidal suspension under acidic conditions overnight and then heating them at 250°C for 12 hr to deposit BOA in the brackets, thus changing the surface free energy of the brackets to reduce bacterial adhesion. Wang et al. [18] prepared blue fluorescent hydrophobic carbon dots by a one-step hydrothermal method using sandalwood alcohol as the carbon source and covalently loaded them onto the surface of an orthodontic bracket with the help of polydopamine, producing an antibacterial effect by generating reactive oxygen species from the positively charged carbon dots, disrupting bacterial cell walls, and damaging genomic DNA. Zeidan et al. [24] used the thermal evaporation method of physical vapor deposition to produce an antibacterial effect by converting zinc oxide particles to gas under high-pressure vacuum heating and spraying them on the surface of the bracket.

However, most current antibacterial and antifouling coatings lack chemical bonding with the substrate surface and have disadvantages such as expensive and complicated manufacturing processes, poor mechanical properties, and biocompatibility when combined with bracket [25]. Polymer-based antimicrobial materials have received extensive attention for their low toxicity, stability, antimicrobial persistence, and ease of modification [26–28]. Compared with monomers, the increase in the relative molecular weight of macromolecules increases the charge density and enhances the adsorption and binding to the surface proteins of bacteria, which in turn enhances the interaction with bacteria and thus achieves bactericidal activity [29]. Antimicrobial groups can be introduced by compounding and modifying polymers, allowing coating materials with high antimicrobial activity to be prepared. Therefore, it is necessary to synthesize a polymeric antimicrobial coating material with adequate mechanical properties, excellent antimicrobial properties, a safe and simple coating process, and good biocompatibility.

Zirconium-based materials are widely used in the medical field because of their high strength, toughness, corrosion resistance, and good biocompatibility. Recently, Chen et al. [29] prepared a hybrid polymer–ceramic antifouling coating based on zirconium-based composite polymer material, which is cross-linked with zirconium epoxide particles and amine-terminated hyperbranched polysiloxane. It has high hardness and adhesion to the substrate and contains amphoteric groups. Hence, the coating material has good oleophobic and antibacterial properties, and the bonding conditions with the substrate are safe, inexpensive, and straightforward. These good properties provide new ideas for ensuring the adhesion of the coating to the bracket and providing long-term antimicrobial capability while maintaining the good mechanical properties of the coating.

In recent years, nanosilver is often used with other antibacterial agents to play a synergistic bactericidal effect, ensure efficient sterilization, and significantly improve biosafety [30–32]. Zhong et al. [33] reported that the presence of the silver nanoparticles could provide additional protection via the silver ion's disinfection toward microbes. Tristán-López et al. [34] reported that silver nanoparticles (AgNPs) can supplement commercial orthodontic adhesives without modifying their mechanical properties with improved bactericidal activity. Eslamian et al. [35] suggested the AgNPs-adhesive showed significant antibacterial activity, which did not change much after 30 days.

Therefore, we aimed to prepare an amine-terminated hyperbranched zirconium–polysiloxane (HPZP)-based coating material. Meanwhile, nanosilver is combined with HPZP coating to test whether adding AgNPs can improve the antibacterial ability of HPZP coating by comparing their structural characterization, biotoxicity, and antibacterial ability. These findings offer a potential foundation for developing new antibacterial stainless-steel brackets to prevent orthodontic complications.

## 2. Materials and Methods

### 2.1. Materials

Zirconium *n*-propoxide, tetrapropyl zirconate (TPOZ), 3-glycidyloxypropyltrimethoxysilane (KH560), 3-aminopropyltriethoxysilane (KH550), (N,N-dimethylaminopropyl) trimethoxysilane (DMASi), 1,3-propane sultone (99%), and dimethyl sulphoxide (DMSO) were procured from Aladdin, China. Acetone and ethanol were purchased from Sinopharm Group, China. Glacial acetic acid (GAA) was purchased from Tianjin Fuyu, China. Cell counting kit-8 (CCK-8), Dulbecco's modified eagle medium (DMEM), fetal bovine serum, 0.25% trypsin solution, phosphate buffered solution (PBS, 0.01 M, pH 7.4), penicillin/streptomycin and artificial saliva were purchased from Solarbio Science & Technology, China. The AgNPs (particle size: 20 *μ*m) were purchased from Zhongkeke You Technology Co, China. The reagents were analytical grades and used as received without additional purification.

### 2.2. Synthesis of Water-Based Zirconium–Siloxane Hybrid Coating

1. Zirconium epoxy particles (ZPs) were synthesized using a reported procedure with slight modifications. In brief, first, DMASi (4.0 g, 19.2 mmol) and 1,3-propane sultone (2.4 g, 19.5 mmol) were dissolved in 20 mL of acetone and stirred for 6 hr at room temperature under nitrogen protection. Then, the reaction mixture was filtered and washed with acetone 3–5 times and dried in a vacuum oven to obtain sulfobetaine silane (SBSi), stored at −20°C for further use. Second, KH560 (1.8 g, 7.6 mmol) and SBSi (0.3 g, 0.9 mmol) were dissolved in 50 mL of deionized water to obtain mixture A. At the same time, TPOZ (1.2 g, 3.6 mmol) was slowly added dropwise into 0.6 g of GAA under a turbo shaker to obtain mixture B. Finally, ZPs were produced by blending mixtures A and B at room temperature for 24 hr.
2. KH550 (20.0 g) was dissolved in the ethanol aqueous solution (90%), and the mixture was stirred at 60°C for 4 hr. Then, amine-terminated hyperbranched polysiloxane (HPSi) was obtained by removing the solvent.
3. The zirconium-siloxane hybrid coating named HPZP was synthesized conveniently by mixing ZPs (3.3 g) and HPSi (1.0 g) at room temperature for 1 hr. The Ag-loaded zirconium–siloxane hybrid coating named HPZP-Ag was prepared by adding 0.065 g (0.15%) AgNPs (20 *μ*m; black; spherical; 99.9% purity) into the HPZP solution [36]. The prepared slurry was kept in a glass bottle at room temperature.

### 2.3. Preparation of HPZP Coating on the Surface of Stainless Steel Sheet

Circular stainless steel sheets with a diameter of 10 mm and a thickness of 1 mm were ultrasonically washed with absolute ethanol and deionized water, respectively. The cleaned stainless steel sheets were obtained by drying them in the oven. Stainless steel sheets with the HPZP and the HPZP-Ag coating, named HPZP@SST and HPZP-Ag@SST, respectively, were prepared using a tetrahedral wet film preparation device (Tang Instrument, 5−10−15−20 *μ*m, China). Uncoated stainless steel sheets were taken as the control group, named stainless steel tank (SST).

### 2.4. Physicochemical Characterizations

#### 2.4.1. Nuclear Magnetic Resonance (NMR) Spectroscopy

SBSi and HPSi were characterized with an NMR spectrometer (Bruker, AVANCE Ⅲ HD 400 MHz) using deuterated methanol as solvent.

#### 2.4.2. Thickness Measurement

The thickness of samples was measured by a thickness gauge (Phynix, Model Surfix, Germany) with an FN 1.5 Sonde probe. Measure the thickness of the SST group, record it as T0, and measure the thickness of the samples and record it as T1. The thickness of each coating is calculated as T1–T0. Three samples were taken from each group; each sample had at least 10 detection points, and the average value was calculated. The investigator was blinded to the experimental groups to perform the evaluation and conducted the statistical analysis.

#### 2.4.3. Scanning Electron Microscope (SEM) Analysis

SEM (TESCAN, VEGA3, Czech Republic) was used to observe the surface morphology of the SST, HPZP@SST, and HPZP-Ag@SST groups.

#### 2.4.4. Contact Angle Detection

Two samples were randomly selected from each group, and the contact angle was detected by an optical contact angle tester (Biolin, Theta, Sweden). At room temperature, a quantitative amount of deionized water (10 *μ*L) was dropped onto the surface of the prepared sample using an automated control system and a probe and left to stand for 40 s to keep the droplet state stable. Then, the software captured dynamic and static images and measured them. For the double-sided contact angle (left contact angle and right contact angle) of the sample, each sample is tested three times, each time not less than three sites. The investigator performed the evaluation and statistical analysis while blinded to the experimental groups.

#### 2.4.5. The Mechanical Stability Test

The mechanical stability of the samples was verified. According to a report by Łyczek et al. [23], we modified the brushing into 100 cycles (200 in total) to check the impact of long-term use of the brackets (Colgate Classic medium-hard toothbrush with Colgate Total dentifrice). After brushing, the brackets were washed with DI water and examined under SEM to evaluate possible damages.

#### 2.4.6. Corrosion Test

To demonstrate the anticorrosion properties of the material in the human oral cavity, the as-prepared samples were soaked in artificial saliva for 9 days, and corrosion and damage were judged by observing the changes in the morphology of the samples before and after immersion of artificial saliva using SEM.

### 2.5. Bacterial Culture

Gram-positive *Streptococcus mutans (S. mutans*), *Staphylococcus aureus (S. aureus*), and Gram-negative *Escherichia coli (E. coli*) were provided by the laboratory of Qingdao Stomatological Hospital. *E. coli* and *S. aureus* were cultivated in Luria–Bertani medium (Sigma–Aldrich, St. Louis, MO). Then, *S. mutans* were incubated in a brain–heart infusion medium (Sigma–Aldrich, St. Louis, MO). 1 × 10^6^ CFU (colony-forming units)/mL bacteria suspension was prepared for later use.

### 2.6. The Antibacterial Ability

#### 2.6.1. CFU Method

The antimicrobial performance of the samples was assessed using the CFU method. SST, HPZP@SST, and HPZP-Ag@SST samples were cocultured with the bacterial suspension (10^6^ CFU/mL, 0.4 mL) for 24 hr. PBS was selected as a negative control. After diluting the sample gradient to 10^−6^, 100 *µ*L of the samples were coated on the medium plates and cocultured for 24 hr before counting. The experiment was repeated three times for each group, and the CFUs on the plate were recorded. The following equation calculates the sterilization results:

(1) $$\begin{array}{c} \text{Bactericidal rate}=\frac{\text{CFU}0-\text{CFU}1}{\text{CFU}0}\times 100\%, \end{array}$$

where CFU0 and CFU1 are the number of colonies in the control and experimental groups, respectively.

#### 2.6.2. Morphology Observed by SEM

Randomly select three samples from SST, HPZP@SST, and HPZP-Ag@SST groups and coculture with the bacterial solution for 24 hr. After cultivation, the bacterial solution was slowly poured out, and the samples were fixed in 2.5% glutaraldehyde solution for 2 hr and gradient dehydrated in 50%, 60%, 70%, 80%, 90%, 100% ethanol solution for 15 min, respectively, and then dried naturally at room temperature. Bacterial adhesion on the surface of each sample was observed by electron microscopy.

### 2.7. In Vitro Biocompatibility of HPZP Coating

The *in vitro* biocompatibility tests were performed using CCK-8 assays. Primary human gingival fibroblasts (hGFs) and human oral keratinocytes (hOKs) cells were derived from Qingdao Stomatological Hospital. The following tests were performed using hGFs and hOKs of the fourth-to-sixth passage. Three samples randomly selected from the HPZP@SST and HPZP-Ag@SST groups were cocultured with DMEM culture solution for 24 hr and filtered through a 0.22 *μ*m microporous membrane filter to obtain the extract. Coculture of cells (2.5 × 10^4^ per well) and different concentrations of samples for 24, 48, and 72 hr and then add fresh CCK-8 reagent to continue to coculture for 1 hr. A microplate reader measured absorbance at an optical wavelength of 450 nm. Cell viability was calculated as follows:

(2) $$\begin{array}{c} \text{Cell viability}=\frac{\text{OD }\left(\text{PC}\right)-\text{OD}\;\left(\text{BK}\right)}{\text{OD}\;\left(\text{NC}\right)-\text{OD}\;\left(\text{BK}\right)}\times 100\%, \end{array}$$

where the PC group refers to adding HPZP@SST or HPZP-Ag@SST immersion solution cocultured with high-sugar DMEM medium into the cells medium, including 100, 200, 400, 800 *μ*L/mL group, while the NC group refers to adding no immersion solution into the cells medium, and blank group (BK) refers to no cell group (background).

### 2.8. Statistical Analysis

All experimental data were shown as the independent experiment's mean ± standard deviation (SD). In addition, a one-way analysis of variance was used for multiple comparisons. All statistics in this experiment were performed in at least three and more parallel experiments. A value of *P* < 0.05 was considered statistically significant.

## 3. Results and Discussion

### 3.1. Synthesis of SBSi and HPSi

**Figure 1:**
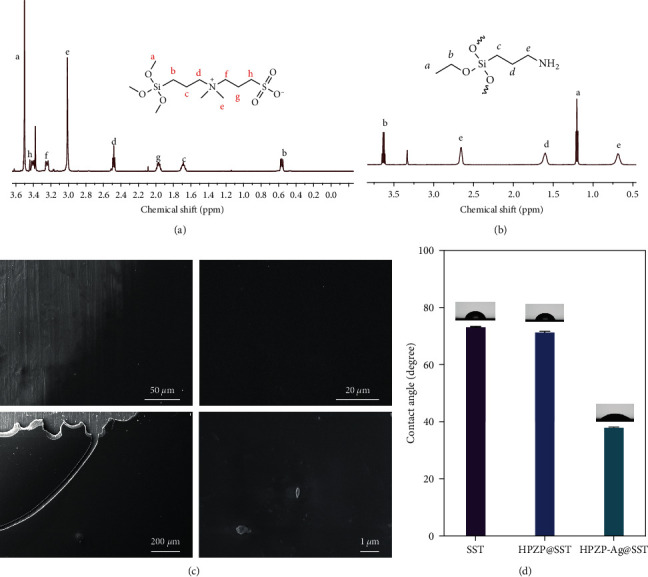
(a) 1H NMR spectrum of SBSi; (b) 1H NMR spectrum of HPSi; (c) SEM images. The coating thickness gradually increases from left to right. The scale was shown in the pictures; (d) WCA of SST, HPZP@SST, and HPZP-Ag@SST. *n* = 3, each sample is tested three times.

NMR is one of the most powerful tools for qualitative analysis of the composition and structure of various organic and inorganic substances; it can also be quantitative analysis and currently plays a key role in explicit structural identification and structural confirmation (qualitative detection) [37, 38]. Figures 1(a) and 1(b) show the samples' 1H NMR spectrum results and corresponding molecular chemical structures. In Figure 1(a), the prepared SBSi has eight characteristic signal peaks, which are marked in the form of a–h, and the chemical shifts corresponding to different distinct signal peaks are various, among which, the singlet at 3.5 ppm corresponds to the splitting of the ─OH in the siloxane group. The triplet peaks at 3.4, 3.15, 2.5, and 0.55 ppm were all obtained by proton coupling near the ─CH_2_ group. The quartets at 1.95 and 1.65 ppm are derived from the coupling of adjacent ─CH_2_ groups at g and c. In Figure 1(b), the prepared HPSi has five characteristic signal peaks marked as a–e. Among them, the NMR peaks at the chemical shift of 0.7 and 2.7 ppm correspond to the groups at c and e, respectively, and the NMR peak at the chemical evolution of 1.6 ppm corresponds to the group at the molecular d. The group at position a exists as a triplet in the NMR spectrum. With the help of the ^1^H NMR spectrum, we confirmed the successful synthesis of SBSi and HPSi.

### 3.2. HPZP Coating and HPZP-Ag Coating

SEM characterized the surface morphology of the materials. As shown in Figure 1(c), the coating is uniformly distributed, dense, and transparent on the stainless steel material under a low-power lens, and the surface is smooth and flat without an obvious granular shape. At high power, the coating still presents a uniform and balanced state. The results showed that the HPZP coating composites prepared by this method are successful. The prepared coating is compact and uniform and can be used for further experiments. After loading AgNPs, Ag particles were dispersed on a coating with a diameter of about 1 *μ*m, as shown in Figure 1(c) under SEM. AgNPs dispersion is not uniform, and the loading is low. AgNPs were largely unstable in the more complex water preparations (MHW) [39], which could partly explain the uneven distribution and larger particle size of AgNPs in HPZP coating, and research showed that aggregation between particles is not conducive to bacteria-killing [40].

### 3.3. Thickness Measurement of Coating

**Figure 2:**
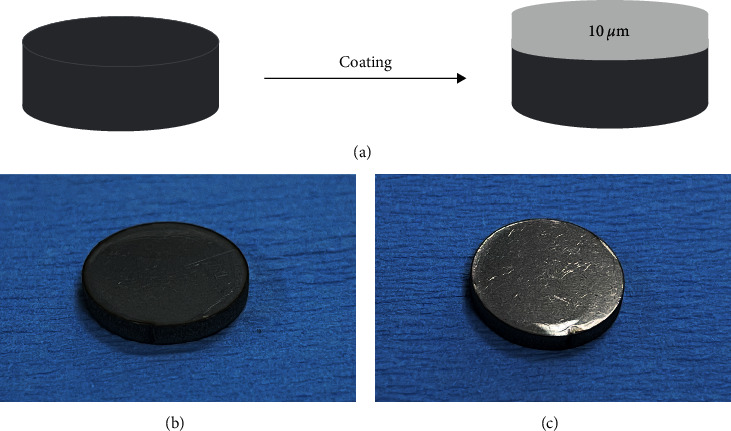
(a) Flowchart of HPZP@SST sample preparation based on tetrahedral wet film preparation process by coating method; (b) SST sample picture; (c) HPZP@SST sample picture.

Figure 2(a) shows the structure diagram of the HPZP@SST sample prepared by the coating process based on the tetrahedral wet film preparer. Figures 2(b) and 2(c) show HPZP@SST sample pictures. The thickness of the coating was measured with a thickness gauge. The test results were averaged three times to reduce error interference, as shown in Table 1. The results showed that the thickness of the coating was about 10 *μ*m, and there was no significant difference between HPZP coating and HPZP-Ag coating (*P* > 0.05). This thickness is thinner than biomimetic calcium phosphate coating on medical grade stainless steel, and Iijima et al. suggested that a coating of 10 *μ*m is esthetic [40, 41]. Therefore, it can be considered that our coating is esthetically pleasing.

### 3.4. Contact Angle Detection

Higher surface hydrophilicity induces more microbial accumulation and reduces the antibacterial ability of the coating [42, 43]. We carried out a water contact angle test experiment to understand the surface-wetting characteristics of different materials. As shown in Figure 1(d), in comparison with the stainless steel sample (73.25° ± 0.18°) and HPZP coating (71.37° ± 0.28°), HPZP-Ag@SST coating (44.62° ± 8.65°) has a notably lower water contact angle (both *P* < 0.001). The results showed that Ag significantly changed the surface wettability and enhanced the hydrophilicity of the material. HPZP@SST was more disadvantageous to cell adhesion and had more vital antibacterial ability than HPZP-Ag@SST.

### 3.5. The Mechanical Stability and Corrosion Resistance of HPZP Coating

**Figure 3:**
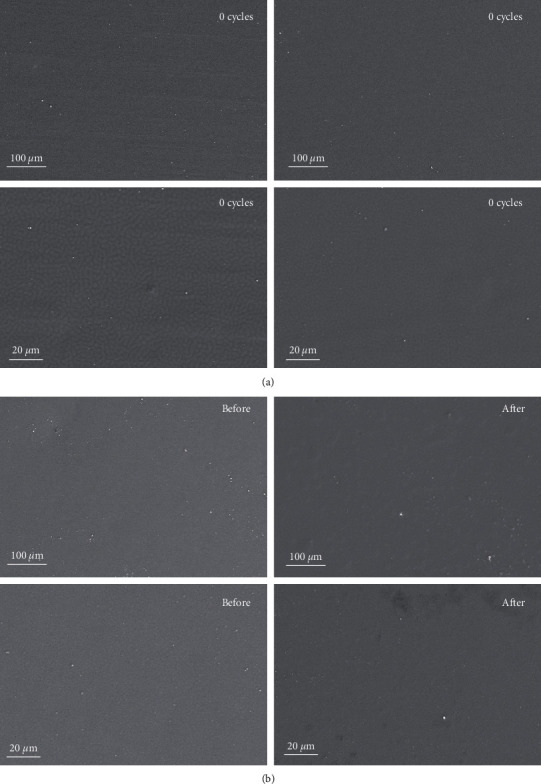
(a) SEM pictures of HPZP@SST before and after 100 cycles of brushing (100 min in total); (b) SEM pictures of HPZP@SST before and after 9 days of immersion in artificial saliva. The scale was shown in the pictures.

As shown in Figures 3(a) and 3(b), after 200 min (100 times of brushing, 2 min each time) simulated brushing and 9 days of artificial saliva immersion, a small amount of peeling appeared on the coating surface, but no obvious defects, indicating that the HPZP coating has a certain degree of mechanical stability and corrosion resistance.

### 3.6. Cytotoxicity Evaluation

**Figure 4:**
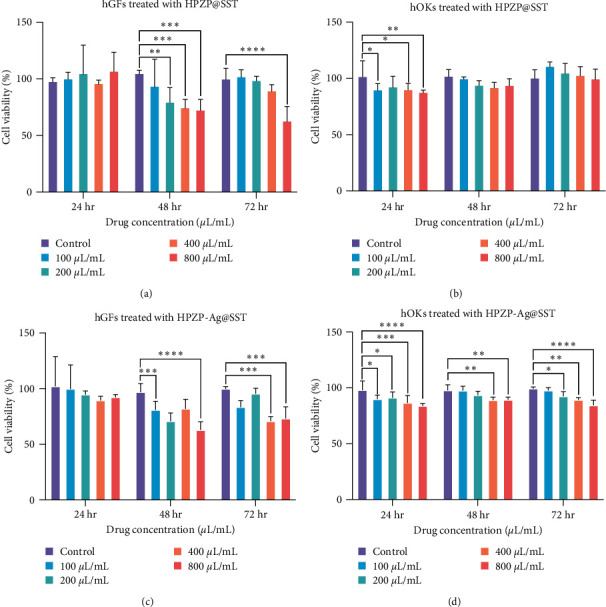
The cytocompatibility of HPZP@SST and HPZP-Ag@SST: (a) cell viability of hGFs treated with different concentrations of HPZP@SST for 24, 48, and 72 hr. *n* = 5; (b) cell viability of hOKs treated with different concentrations of HPZP@SST for 24, 48, and 72 hr. *n* = 5; (c) cell viability of hGFs treated with different concentrations of HPZP-Ag@SST for 24, 48, and 72 hr. *n* = 5; (d) cell viability of hOKs treated with different concentrations of HPZP-Ag@SST for 24, 48, and 72 hr. *n* = 5. Data are presented as the mean ± SD.  ^*∗*^*P* < 0.1,  ^*∗∗*^*P* < 0.01,  ^*∗∗∗*^*P* < 0.001,  ^*∗∗∗∗*^*P* < 0.0001 vs. control group.

hOKs and hGFs are often used in in vitro studies of the oral cavity [44, 45]. The performance of the coating is closely related to these two types of cells because the coating is in direct contact with both types of cells in the mouth. Here, we chose these two kinds of cells to conduct in vitro experiments to simulate the coating applied to the oral cavity. The results of CCK-8 detection are shown in Figure 4. We added the immersion liquid of HPZP@SST or HPZP-Ag@SST cocultured with DMEM high-glucose medium into the cell culture medium according to a concentration gradient of 0, 100, 200, 400, 800 *μ*L/mL, respectively. As shown in Figure 4(a), after 24, 48, and 72 hr, the survival average rate of hGFs cells cocultured with different concentrations of HPZP@SST soaking solution was 101.3%, 85.3%, 90.8%, respectively. The cell survival rate of HPZP-Ag@SST is similar to that of the HPZP@SST group (Figure 4(c)), where the HPZP-Ag@SST group was slightly more cytotoxic than the HPZP@SST group. The results showed that the higher the concentration of HPZP@SST or HPZP-Ag@SST, the more inhibited the viability of hGFs cells. The viability of hGFs cells was the lowest after 48 hr, and hGFs increased a little after 72 hr. When HPZP@SST or HPZP-Ag@SST soaking solution was cocultured with hGF cells, the concentration of 800 *μ*L/mL seemed to be more toxic to hGF cells than other concentration groups.  Figure 4(b) shows the survival rate of hOKs cells cocultured with HPZP@SST soaking solution after 24, 48, and 72 hr. The concentration of the soaking solution increased, but the toxicity to the cells did not increase significantly, and the cells proliferated as time went on. As shown in Figure 4(d), after 24, 48, and 72 hr, the average survival rate of hOKs cells cocultured with HPZP-Ag@SST soaking solution were 89.9%, 93.4%, and 92.6%, respectively. There was a positive correlation between the concentration of the soaking solution and the cytotoxicity, and each concentration inhibited the cell viability, but there was no significant change with time. Hu et al. [46] reported that viability higher than 80% is normally acceptable for biomaterials with good biocompatibility. The above results showed that the toxicity of HPZP coating and HPZP-Ag coating to cells is relatively slight and has certain biocompatibility, and the toxicity of HPZP-Ag coating is greater than that of HPZP coating.

### 3.7. Antibacterial Results

**Figure 5:**
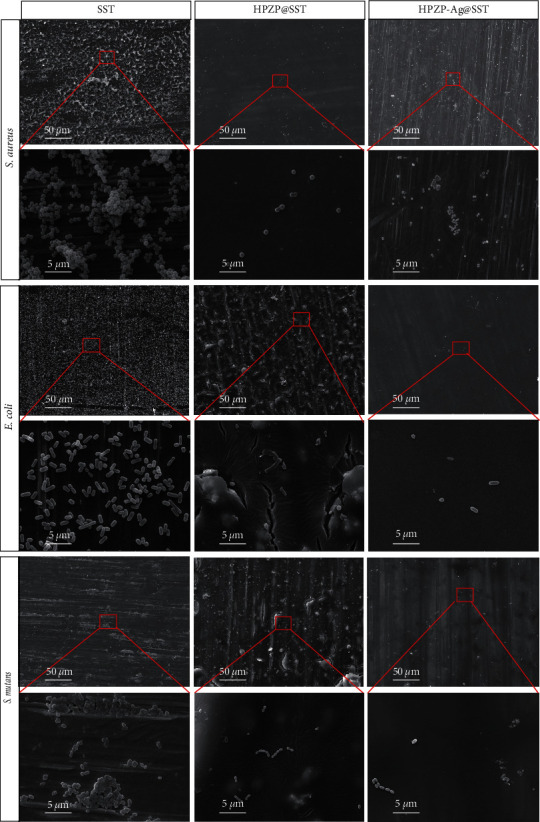
SEM pictures of HPZP@SST and HPZP-Ag@SST after cocultured with *S. aureus*, *E. coli*, and *S. mutans* for 24 hr. The scale was shown in the pictures.

**Figure 6:**
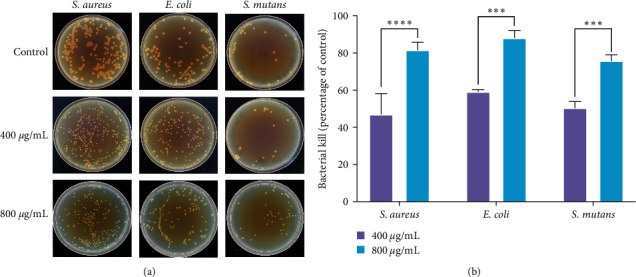
The antibacterial properties of HPZP@SST: (a) effect of colony formation on *S. aureus*, *E. coli*, and *S. mutans* in plate after treating with different concentrations of HPZP@SST for 24 hr; (b) the bactericidal rate of the HPZP@SST on *S. aureus*, *E. coli*, and *S. mutans*. *n* = 3. Data are presented as the mean ± SD.  ^*∗∗∗*^*P* < 0.001,  ^*∗∗∗∗*^*P* < 0.0001, 800 vs. 400 *μ*g/mL group.

Subsequently, *S. aureus*, *E. coli*, and *S. mutans* were cocultured with SST, HPZP@SST, and HPZP-Ag@SST for 24 hr. As shown in Figure 5, changes in the number and morphology of bacteria were observed by SEM. As shown in Figure 6, after different materials were cocultured with different bacteria, the number of bacteria increased from small to large: HPZP@SST < HPZP-Ag@SST < SST. The surface of HPZP@SST was smooth, indicating all the bacteria had been killed. In contrast, the three types of bacteria on the surface of SST were the most abundant. For the HPZP coating loaded with AgNPs, its resistance to *S. aureus* was stronger, while its resistance to the other two bacteria was weaker. As shown in Figure 5, the bactericidal effects of different materials are as follows: SST < HPZP-Ag@SST < HPZP@SST. The antibacterial properties of HPZP@SST on *S. aureus*, *E. coli*, and *S. mutans* for 24 hr are shown in Figure 6, with an antibacterial rate of nearly 80%. HPZP@SST was cocultured with *S. aureus*, *E. coli*, and *S. mutans*, respectively, and the bactericidal ability of the 800 *μ*g/mL group was stronger than that of the 400 *μ*g/mL group (all *P* < 0.05). Our results showed that HPZP coating has a stronger bactericidal effect than HPZP-Ag coating, which is inconsistent with the report of Zhou et al. [47]. Possible reasons may be the uneven dispersion and the low loading of AgNPs in the coating. AgNPs were largely unstable in the more complex water preparations (MHW) [39], and the aggregation between particles is not conducive to sterilization [40].

## 4. Conclusions

A new HPZP coating with good mechanical stability, antibacterial adhesion, as well as biocompatibility was designed and synthesized. The HPZP coating has great potential for preventing complications in the orthodontic process based on the following factors: (1) HPZP coating has a certain degree of mechanical stability and corrosion resistance, (2) the CCK8 test confirmed that the HPZP coating exhibited low cell toxicity, with higher than 80% viability after 24 and 72 hr cocultured with hGFs or hOKs, and (3) HPZP coating exhibited a comparable antibacterial effect with an antibacterial rate of nearly 80%. Some limitations of this work have to be addressed: (1) we did not deeply explore the reasons for the degradation of bacteriostatic performance of HPZP coating caused by Ag, (2) the coating was simulated on smooth and flat steel sheets and was not applied on the actual uneven tooth brackets, which was caused by the limitations of the experimental method, and (3) no *in vivo* studies were conducted. Further studies will be considered to further explore the function of HPZP coatings. Based on the above, therefore, we will continue to improve our work further. In summary, the HPZP coating is an effective antibacterial and biocompatible coating that has the potential to be applied for the management of the orthodontic process.

## Figures and Tables

**Table 1:** Film thickness measurement using a thickness gauge.

Group	Thickness (*μ*m)			Mean ± SD
SST	9.9	10.4	9.6	9.97 ± 0.40
HPZP@SST	10.3	9.8	10.2	10.2 ± 0.26

## Data Availability

Data will be available on request.
